# Nonlinear seepage erosion model of water inrush considering particle size distribution of karst collapse column and its engineering applications

**DOI:** 10.1038/s41598-022-21623-4

**Published:** 2022-10-12

**Authors:** Bin Yang, Wenhao Shi, Xin Yang

**Affiliations:** 1 School of Civil Engineering, University of Science and Technology Liaoning, Anshan, 114051 China; 2 School of Civil Engineering, Suzhou University of Science and Technology, Suzhou, 215009 China; 3 School of Civil and Transportation Engineering, Guangdong University of Technology, Guangzhou, 510006 China

**Keywords:** Civil engineering, Environmental impact

## Abstract

Water inrush through karst collapse column is one of the great disasters which threaten coal mine safety production. The particle size distribution of karst collapse column is one of its most basic physical properties, which has a strong correlation with particle migration, and is an important basis for evaluating the water inrush risk of collapse column. The nonlinear flow tests of broken rock under different gradation conditions were carried out by a custom-built apparatus, and the relationship equation between nonlinear flow parameters (permeability and non-Darcy factor) and Talbol power exponent *n* were constructed. A nonlinear flow model with variable mass of water inrush from karst collapse column was established. The spatio-temporal evolution law of pressure, velocity, porosity and concentration under particle loss and the influence of particle gradation on the water inrush risk of karst collapse column at Fan gezhuang mine were discussed. During the water inrush, the flow state of fluids in karst collapse column gradually transitions from a weak inertial flow to a strong one, eventually becoming a turbulent flow. The flow model based on single flow state cannot reflect the essence of flow regime transition in water inrush. The larger *n* is, the stronger the water permeability of the karst collapse column, the faster the particles migrate and are lost, the faster the flow channel with high porosity develops, the shorter the time for the water inflow to reach its peak value, and the greater the risk of water inrush.

## Introduction

Karst collapse columns, a unique hidden vertical structure found in the coalfields of the Boreal Permo-Carboniferous system in China, are widely distributed in 45 coal mining areas of 20 coal fields^1^. The bottom of karst collapse columns is situated in a cave of soluble rocks. Generally, it may serve as a strong water-conducting channel that not only transmits hydraulic connections between an aquifer and a working face of an Ordovician limestone but also induces water inrush. For example, the ‘3·1’ major water inrush accident that took place in the Camel Hill Mine in Wuhai City, Inner Mongolia, on 1 March 2010, caused 32 deaths, 7 injuries and a direct economic loss of RMB 48.53 million^2^. On 10 September 2018, a water inrush accident from the seam floor of the 1313 coal face occurred in the Xiaoyun coal mine in Shandong Province. During this accident, the peak inrush water range reached 3,673 m^3^/h, resulting in a direct economic loss of up to RMB 25.66 million^3^.

Scholars at home and abroad have extensively investigated karst collapse column water inrush. Wang^4^ investigated the water inrush rules of karst collapse columns at the floor and the coal passing stratum by developing a laboratory table for water-conducting karst collapse column water inrush simulation. Zhang^5^ designed a three-dimensional large-sized simulation experimental model to reproduce the karst collapse column water inrush situation in Luotuoshan coal mine and acquire both the clinical hydraulic pressure and points of water inrush. Karst collapse column water inrush has rarely been studied using similarity simulation experiments, but the evolution law of non-linear seepage parameters has been frequently investigated in relation to fractured rock masses. For example, Moutsopulos^6^ discovered that both linear and non-linear resistance terms in the Forchheimer equation are inclined to decrease as large particle contents in porous media increase using non-linear seepage tests for accumulative granular porous media. Neild^7^ concluded that non-linear seepage parameters are sensitive to changes in pore structures based on research findings obtained by Dupuit and Forchheimer. The process of fractured rock mass seepage is usually combined with changes in the pore structure. Bai^8^, Yao^9^, Chen^10^, and Ma^11,12^ investigated variable mass flows of fractured rock masses in line with Darcy’s law. The impacts of variable mass flows and particle size distribution of fractured rock masses on non-linear seepage parameters under non-linear flow conditions have rarely been reported.

In the numerical simulation, Huang^13^ not only used Darcy’s law to describe the seepage behaviour of rock masses, but they also constructed a rock stress-rock damage-seepage coupling model based on the relationship between rock mass damages and permeability. On this basis, the influence of development height and water pressure of concealed collapse column on coal floor water inrush was studied. Yao^14^ constructed a mechanical model for deformations, seepage and erosional forces incurred by karst collapse column water inrush under multi-field coupling conditions by generally considering rock skeleton deformation, water seepage and filling particle migration during the seepage of karst collapse columns. Nevertheless, their model does not manifest non-linear seepage characteristics. Zhao^15^ built a model that integrates non-linear coupling seepage and conduit flows into one by combining a fluid–structure coupling theory, a flow state conversion theory and a strength-reduction approach of rock masses in order to analyse flow state conversions of bursting water in pressure-bearing caves. And highlighted the importance of whole-process analysis on flow state variations and water inrush to reveal the seepage-inducing water inrush mechanism. The author’s team^16–18^ investigated uniform dynamic characteristics for three flow fields, namely water-bearing strata, water-conducting channels and roadway, from the perspective of flow state conversion. On this basis, Yang^19^ proposed a flow erosion model of water inrush in a fractured zone in the Jiangjiawan Mine, that couples the Darcy, Forchheimer, and Navier–Stokes fields under the theory of continuum mechanics, and the effects of rock disintegration and the coupled effects of flow and erosion were incorporated. However, the above model does not consider the influence of karst collapse column particle size distribution on water inrush.

In summary, there are two flaws in the existing literature on the disastrous mechanism of karst collapse column seepage. First, the sensitivity of non-linear seepage parameters to particle size distribution remains unclear; second, the flow state conversion mechanism caused by particle migration during the non-linear seepage of karst collapse column water inrush is still unknown. Therefore, a non-linear seepage testing system for porous media independently developed at the Northeastern University was used in this study to perform non-linear seepage tests on fractured rock masses under different particle size distribution conditions. An equation that expresses the relationship between non-linear seepage parameters and the Talbol power exponent was established in this manner. Thereafter, a non-linear seepage model was constructed for the variable mass of karst collapse column water inrush to investigate the influence of rapid and non-linear seepages and particle size distribution on the dangers of karst collapse column water inrush caused by particle loss. Finally, the disastrous mechanism of water inrush caused by karst collapse column seepage was revealed, providing a research foundation for early warning, prevention and control of water inrush in mines, as well as reasonable prediction of the water inrush range, explorations of the evolvement characteristics of seepage fields under complex hydrogeological conditions, etc.

## Relationship between non-linear seepage parameters and particle size distribution

### Experimental apparatus

**Figure 1:**
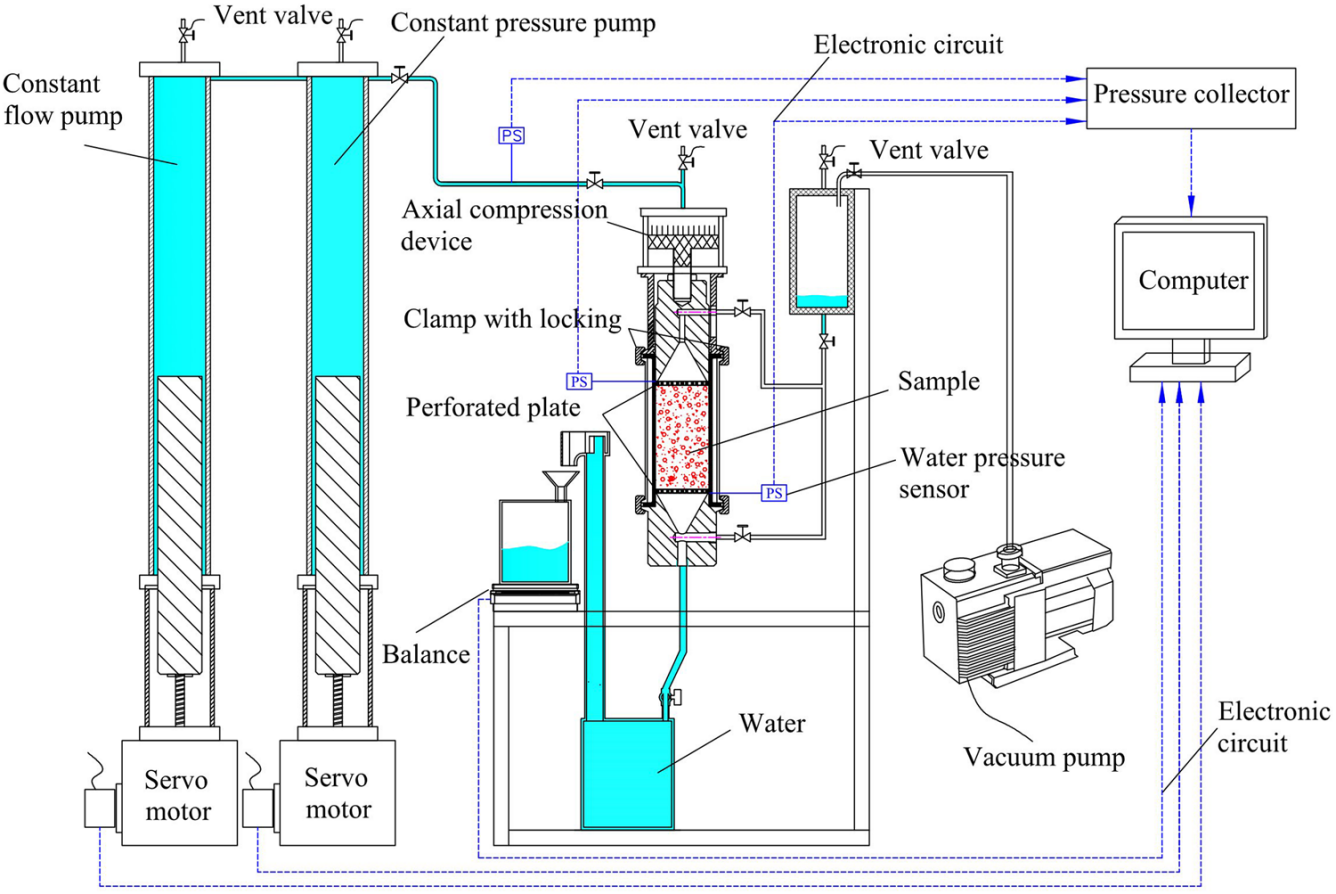
Experimental schematic diagram.

The experimental apparatus was performed at Northeastern University, China, used a custom-built apparatus to model high-velocity seepage in a porous medium^20^. It mainly consists of four parts: the experimental unit, the water supply system, the data measurement equipment, and the recording equipment. Figure 1 illustrates the system connections and principles. Solid blue represents water, red parts represent samples, and blue dashed lines represent circuits.

### Experimental scheme and method

Karst collapse columns contain internally fractured rock masses of various grain sizes, which form a high-dimensional parameter space. To investigate the influence of particle size distribution on permeability and the non-Darcy factors, the Talbol gradation theory was selected to describe the size distribution of fillings in karst collapse columns^21^:

1 $$p_{i} = \left\{ \begin{aligned} & \left[ {\left( {\frac{{d_{i} }}{{d_{m} }}} \right)^{n} - \left( {\frac{{d_{i - 1} }}{{d_{m} }}} \right)^{n} } \right] \times 100\% \;\; \left( {i > 1} \right) \hfill \\ & \left( {\frac{{d_{i} }}{{d_{m} }}} \right)^{n} \times 100\% \;\;\left( {i = 1} \right) \hfill \\ \end{aligned} \right.$$

where *p*_*i*_ is the mass fraction of particles in a particle size range of group *i* (%), *d*_*i*_ is the particle diameter (mm), *d*_*m*_ is the maximum particle diameter (mm) and *n* is the Talbol power exponent, which is dimensionless.

To reduce the influence of the size effect of particles on the experimental results, the maximum diameter of particulate matter should be less than 1/5–1/6 of D, which is the inner diameter of the sample-loading barrel used in the experiment. Since *D* = 60 mm, the maximum particle size of the selected sand grains must be less than 10 mm. In Eq. (1), the Talbol power exponent of the test samples with five different particle size distributions was set at 0.5, 0.75, 1.0, 1.25 and 1.5. Table 1 shows the mass fractions of particles of various size ranges.

**Table 1:** Grain size distributions of samples.

*d* (mm)	*p*_*i*_ (%)				
	*n* = 0.5	*n* = 0.75	*n* = 1.0	*n* = 1.25	*n* = 1.5
0–0.075	7.25	1.95	0.53	0.14	0.04
0.075–0.15	5.31	2.50	1.05	0.42	0.16
0.15–0.3	5.20	3.04	1.58	0.77	0.36
0.3–0.6	7.36	5.11	3.16	1.83	1.03
0.6–1	8.38	6.80	4.91	3.33	2.18
1–2	12.37	11.68	9.82	7.76	5.90
2–2.36	9.43	10.06	9.54	8.50	7.26
2.36–4.75	15.32	18.23	19.30	19.18	18.32
4.75–9.5	29.36	40.63	50.11	58.07	64.76

The test tube (height of 0.2 m, diameter of 0.06 m and wall thickness of 0.007 m) was made of organic glass, which permitted the visualization of the test process. During filling, particles should be filled by layers and compacted uniformly by a mallet to ensure the uniformity of the sample. Before starting the experiments, the vacuum pump was used to remove the air from the inside of the sample, and then the negative pressure in the test tube is used to slowly passed the water through the tube from the bottom to the top. This method can make the sample saturation up to 95%, effectively avoid the influence of air on the experimental results.

Water was pumped from the bottom of the experimental column, which flowed upwards through the test tube, and then flowed into the mass measurement device. The quality method was adopted for measuring flow rate experimentally. Three experiments were performed under each pressure condition, and the average value of the experimental results was used for analysis. A total of 5 samples, about 180 experiments need to be conducted. The water pressure upstream and downstream of the sample was measured by a pressure collector. The real-time experimental data were recording by a data collection software in the computer. It should be noted that, the measurements were carried out after the flow stabilized for at least 1 min.

### Experimental results

**Figure 2:**
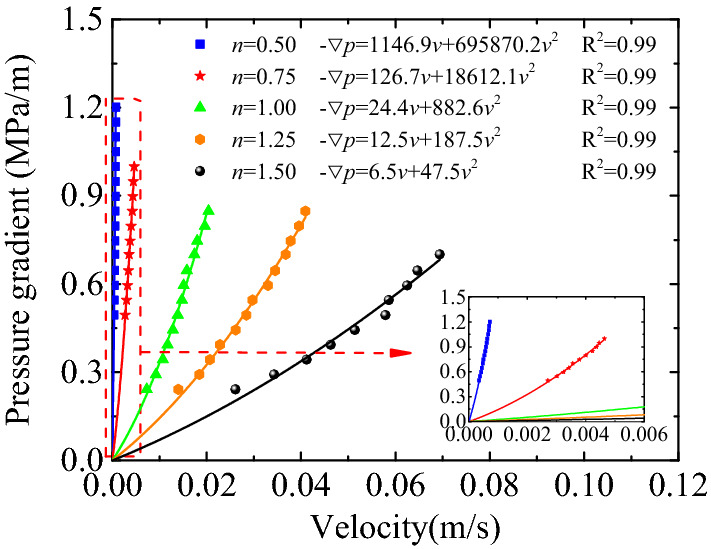
The relationship between pressure gradient and flow velocity under different power exponent of Talbol.

The corresponding relationship between the pressure gradient and flow rate of the samples with five different particle size distributions was obtained. Thereafter, data fitting was completed using the Forchheimer equation, as shown in Fig. 2. The result of the corresponding relationship fitting was satisfactory, and *R*^2^ = 0.99 in all cases. For the same sample, the pressure gradient tended to grow non-linearly as the flow rate gradually increased, and a larger slope of the curve indicates that the non-linearity of the pressure gradient and flow rate is more apparent. When the pressure gradient changed within a range of 0–1.2 MPa/m and the Talbol power exponent increased, the superior fovea of the corresponding curves became more prominent. This indicates that porous media with a relatively high Talbol power exponent are likely to produce non-linear seepages when subjected to the same pressure gradient.

**Figure 3:**
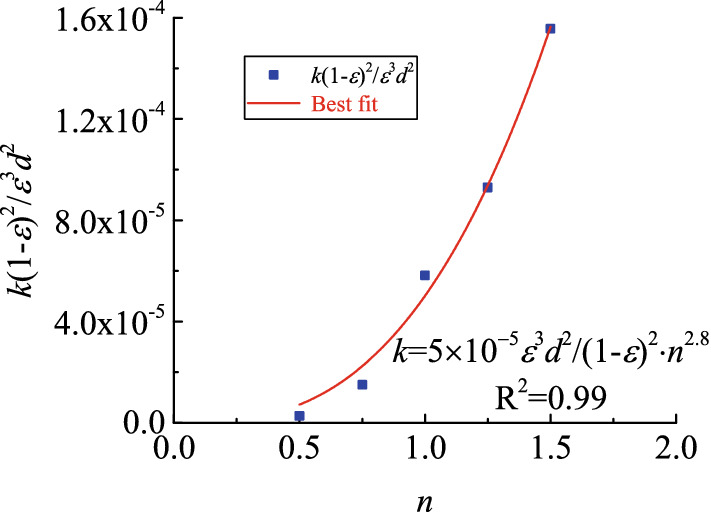
Variation curve of $\frac{{k(1 - \varepsilon )^{2} }}{{\varepsilon^{3} d^{2} }}$ with *n.*

**Figure 4:**
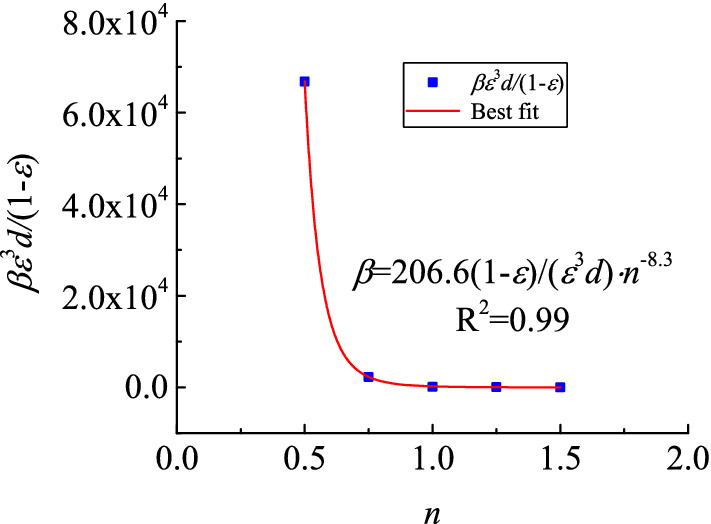
Variation curve of $\frac{{\beta \varepsilon^{3} d}}{1 - \varepsilon }$ with *n.*

The porosity of the five samples was 0.28. This phenomenon simply means that the pores of samples of the same volume have the same total volume. The pores of particles in the sample had diverse forms because of differences in particle size distributions, further resulting in uncertainties in seepage paths and resistance levels of fluids in the sample. As shown in Figs. 3 and 4, non-linear seepage parameters may change along with the Talbol power exponent under corresponding testing conditions. As shown in these figures, the corresponding permeability increased by two orders of magnitudes, from a magnitude of 10^−12^ to 10^−10^, when the Talbol power exponent increased from 0.5 to 1.5. In terms of the non-Darcy factors, they decreased by four orders of magnitudes, from 10^8^ to 10^4^. As the Talbol power exponent increased, the rate of increase in permeability and the non-Darcy factors increased and decreased, respectively. As shown in Table 1, as the Talbol power exponent increased, the large and small particle contents increased and decreased, respectively. When the total porosity of the samples was the same, porous media formed by small particle accumulation had a large number of pores and a low mean pore diameter. However, porous media formed by large particle accumulation had a few pores and a large average pore diameter. Based on the comparison, the latter had a low tortuosity, which shortened the path required by lateral diversions of fluids and reduced flow resistance.

Scholars have extensively investigated the values selected for permeability *k* and the non-Darcy factor *β*, as presented in Table 2. Both the non-Darcy factor and permeability are inherent attributes of a medium. When the particle size gradation of samples change, the compositions of particles in the sample may become diverse and uncertain. There are no equations in Table 2 that can express the relationship between permeability, non-Darcy factor and particle size distribution. Thus, the Talbol power exponent was introduced to slightly modify and improve them.

2 $$k = \alpha_{1} \frac{{\varepsilon^{3} }}{{(1 - \varepsilon )^{2} }}\overline{d}^{2} n^{{\alpha_{2} }}$$

3 $$\beta = \alpha_{3} \frac{(1 - \varepsilon )}{{\varepsilon^{3} \overline{d} }}n^{{\alpha_{4} }}$$

**Table 2:** The empirical expression of permeability and non-Darcy factor^20^.

Author (year)	Permeability *k* (m^2^)	Non-Darcy factor *β* (m^−1^)
Ergun (1952)	$k = \frac{{\varepsilon^{3} \cdot d^{2} }}{{150(1 - \varepsilon )^{2} }}$	$\beta = \frac{1.75(1 - \varepsilon )}{{\varepsilon^{3} \cdot d}}$
Irmay (1964)	$k = \frac{{\varepsilon^{3} \cdot d^{2} }}{{180(1 - \varepsilon )^{2} }}$	$\beta = \frac{0.6(1 - \varepsilon )}{{\varepsilon^{3} \cdot d}}$
Kovács (1981)	$k = \frac{{\varepsilon^{3} \cdot d^{2} }}{{144(1 - \varepsilon )^{2} }}$	$\beta = \frac{2.4(1 - \varepsilon )}{{\varepsilon^{3} \cdot d}}$
Fand (1990)	$k = \frac{{\varepsilon^{3} \cdot d^{2} }}{{214(1 - \varepsilon )^{2} \left[ {1 + \frac{2}{3}\frac{d}{{D\left( {1 - \varepsilon } \right)}}} \right]^{2} }}$	$\beta = \frac{1.57(1 - \varepsilon )}{{\varepsilon^{3} \cdot d}}\left[ {1 + \frac{2}{3}\frac{d}{D(1 - \varepsilon )}} \right]$
Kadlec (1996)	$k = \frac{{\varepsilon^{3.7} \cdot d^{2} }}{255(1 - \varepsilon )}$	$\beta = \frac{2(1 - \varepsilon )}{{\varepsilon^{3} \cdot d}}$

where *k* is the permeability (m^2^), *β* is the non-Darcy factor (m^−1^), *ε* is the porosity, which is dimensionless, $\overline{d}$ is the mean particle size (m) and *α*_1_, *α*_2_, *α*_3_ and *α*_4_ are the fitting coefficients obtained by fitting the experimental data.

Equations (2) and (3) were used to find the corresponding relationship between the non-linear seepage parameter and the Talbol power exponent via data fitting (see Figs. 3 and 4). Here, the correlation coefficient (R^2^) was 0.99 in all cases, and the *α*_1_, *α*_2_, *α*_3_ and *α*_4_ values were 5 × 10^−5^, 2.8, 206.6 and − 8.3, respectively.

## Non-linear seepage model for the variable mass

### Basic assumptions

1. A karst collapse column is composed of three phases: an aqueous phase, a rock skeleton phase and a movable fine particle phase.
2. The flow rates of movable fine particles and the water phase are identical at any moment, and they move together, neglecting flow velocity losses and energy loss caused by particle collisions during water flows carrying sand.
3. The rock skeleton phase in a karst collapse column is rigid; the skeleton remains unchanged during the loss of movable fine particles, and both water and the water–sand mixed fluid are incompressible single-phase Newtonian fluids.
4. The porosity of the model remains effective; in other words, connected pores in a karst collapse column are completely filled with the water and movable fine particle phases, and pores that are not connected are viewed as the skeleton of this column.
5. The model-solving area is completely saturated.

### The fluid control equation for Ordovician limestone aquifers

1. The permeability of Ordovician limestone aquifers is generally low, and the pressure gradient has a linear relationship with the flow velocity, and the seepage itself conforms to Darcy’s law, which can be expressed as the equation below:
  4 $$v_{D} = - \frac{{k_{D} }}{{\mu_{w} }}\left( {\nabla P_{D} - \rho_{w} g} \right)$$
  where *k*_D_ represents the permeability of the Ordovician limestone aquifer (m^2^), *μ*_w_ is the coefficient of dynamic viscosity of water (Pa·s), *v*_*D*_ is the velocity (m/s), *P*_*D*_ is the pressure (Pa), *g* is the gravitational acceleration (m/s^2^) and *ρ*_w_ is the water density (kg/m^3^), *D* is only the subscript, representing the Darcy field.
2. The equation of continuity for non-steady seepages is as follows:
  5 $$\frac{{\partial \left( {\rho_{w} \varepsilon } \right)}}{\partial t} + \nabla \cdot \left( {\rho_{w} v_{D} } \right) = q\rho_{w}$$
  where *t* is the time (s), and *q* is the source (sink) strength. The *q* values were set to be positive and negative for the sources and sinks, respectively (s^−1^). In the case of passive sink flows, there exists *q* = 0. Moreover, ∇ is the divergence operator.

### The fluid control equation for mixed fluids in the water-conducting channels of karst collapse columns

1. The equation of continuity for mixed fluids
  Seepage produced by karst collapse column water inrush has a very complicated mechanism. It is a mass-variable non-linear seepage^22^. Provided that water–sand mixed fluids are regarded as the incompressible single-phase Newtonian fluids, the equation of continuity can be written as follows^19^:
  6 $$\frac{{\partial \rho_{m} \varepsilon }}{\partial t} + \nabla \cdot \left( {\rho_{m} v_{F} } \right) = \rho_{s} \frac{\partial \varepsilon }{{\partial t}}$$
  where *ρ*_m_ is the density of a mixture composed of migrated particles and water in the channel at moment *t* (kg/m^3^), and *ρ*_s_ is the density of solid particles (kg/m^3^), *F* is only the subscript, representing the Forchheimer field.
2. The equation of motion for mixed fluids
  7 $$- \nabla P_{F} = \frac{{\mu_{m} }}{k}v_{F} + \beta \rho_{m} v_{F}^{2}$$
  where *μ*_m_ is the dynamic viscosity of water–sand mixed fluids (Pa s).
3. A porosity evolution equation
  8 $$\left\{ \begin{aligned} & \frac{\partial \varepsilon }{{\partial t}} = \lambda \left( {\varepsilon_{\max } - \varepsilon } \right)c_{F} v_{F} \, v_{F} > v_{Fc} \\ & \frac{\partial \varepsilon }{{\partial t}} = 0 \, v_{F} \le v_{Fc} \\ \end{aligned} \right.$$
  where λ is the suffusion coefficient (m^−1^), and its dimension is a reciprocal value of length, which can be obtained through laboratory tests. In addition, *v*_*Fc*_ is the critical flow velocity of particle initiation (m/s), and *ε*_max_ represents the maximum porosity.
4. The concentration transmission equation for migrated particles.
  Assume that the mixed fluids are incompressible. Furthermore, the diffusion effects of particles in mixed fluids are ignored. The concentration transmission equation for migrated particles was obtained using the law of conservation of mass of fluids and the conventional constitutive equation of seepage suffusion^23–25^:
  9 $$\frac{{\partial \left( {c_{F} \varepsilon } \right)}}{\partial t} + \nabla \cdot \left( {c_{F} v_{F} } \right) = \frac{\partial \varepsilon }{{\partial t}}$$
  where *c*_*F*_ is the concentration of migrated particles at moment *t* or the volume fraction of solid particles in mixed fluids.
5. The equation for changes in the density of mixed fluids
  10 $$\rho_{{\text{m}}} = \left( {1 - c_{F} } \right)\rho_{{\text{w}}} + c_{F} \rho_{{\text{s}}}$$
6. The relationship between the dynamic viscosities of mixed fluids and water^26^
  11 $$\mu_{m} = \mu_{w} \left( {1 - c_{F} } \right)^{ - 2.5}$$
  Equations (2) and (3) were used to describe the relationship between permeability, non-Darcy factor and particle gradation of the karst collapse column fillings. Equations (2), (3), (8)–(11) are all auxiliary equations.

### The control equation for mixed fluids in roadways

1. The advection–dispersion equation
  The transport properties of particles in a roadway can be described using the advection–dispersion equation below^27^:
  12 $$\frac{{\partial c_{N} }}{\partial t} = K_{dis} \nabla^{2} c_{N} - v_{N} \nabla \cdot c_{N} - c_{N} K_{dep}$$
  where *K*_dep_ and *K*_dis_ are the sedimentation and dispersion coefficients, respectively (s^−1^). Since mixed fluids move rapidly in a roadway, the corresponding diffusion effects are insignificant when compared to those of advection, and *K*_dis_ = 0. *N* is only the subscript, representing the Navier–Stokes field.
2. Navier–Stokes equations
  13 $$\rho_{m} \frac{{\partial v_{N} }}{\partial t} + \left( {\rho_{m} v_{N} \cdot \nabla } \right)v_{N} + \nabla P_{N} = \mu_{m} \nabla^{2} v_{N} + \rho_{m} g$$
3. The continuity equation for mixed fluids
  14 $$\frac{{\partial \rho_{m} }}{\partial t} + \nabla \cdot \left( {\rho_{m} v_{N} } \right) = 0$$

### Numerical solutions to the FELAC2.2-based model

Neighbouring fields must meet three conditions in order to combine three flow fields: pressure balance, velocity continuity and concentration continuity. The model can be solved if the Forchheimer equation set is closed. There are 8 uncertain model parameters in Eqs. (2), (3), (6)–(11): *P*_*F*_, *v*_*F*_, *c*_*F*_, *ε*, *k*, *β*, *ρ*_*F*_ and *μ*_*m*_. The system of equation is sufficient to solve all the equation, and properly determine the values for the 8 uncertain model parameters. A weak form of fluid mechanics equation was constructed based on the virtual displacement principle using the FELAC2.2 software (Finite Element Language And it’s Compiler). The convective term was dispersed using a finite volume method, while the remaining terms were dispersed using a finite element method that was performed to numerically solve the model. For details, please refer to the literatures Ref.^19^ and Ref.^28^.

## Case studies on karst collapse column water inrush

### Project overview

**Figure 5:**
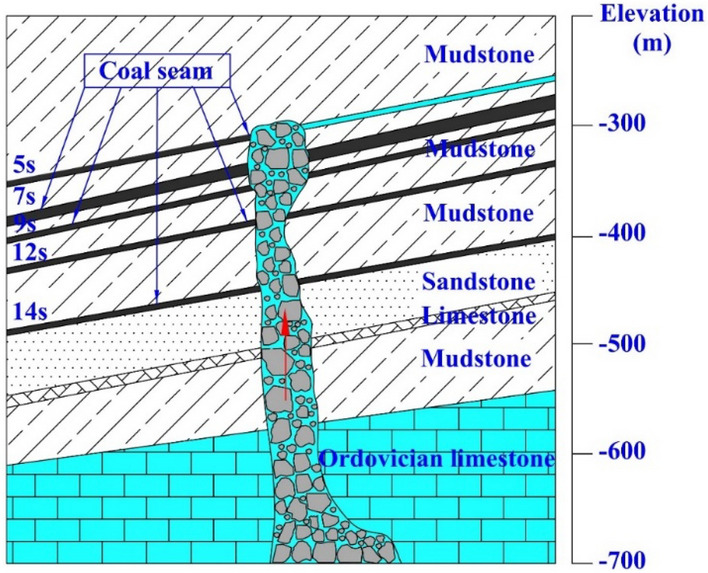
The location and spatial distribution of the Karst collapse column.

The Fangezhuang Mine is located to the southeast of the Kaiping Coalfield in Tangshan City, Hebei Province. Its stratum is oriented NE–SW, inclining towards NW. Furthermore, the corresponding stratigraphic dip is generally within a range of 8°–24°. Folds and fractures are developed in this area. According to the structural characteristics, the minefield is divided into 3 structural areas, namely Tatuo syncline area in the north, central monocline structural area and Bigezhuang syncline area in the south^29^. On 2 June 1984, a catastrophic water inrush from the karst collapse column of the Ordovician limestone occurred at the 2171 working face of the Kailuan Fangezhuang Mine. This is rare in the history of mining. According to calculations based on the flooding volume, the average capacity of water inrush was up to 2053 m^3^/min in peak hours. The water inrush channels of the 2171 working face have been proven to be Ordovician limestone karst collapse columns that have an extremely strong water-conducting capability and are hidden within the coal face based on the characteristics of the water inrush procedure and capacity, as well as data collected from extensive drilling, geophysical prospecting and hydrogeological tests, etc., during water control^30^. Figure 5 depicts the position and spatial forms of the karst karst collapse columns. The total volume of the karst collapse columns is 861,000 m^3^. There is a large cave (volume: 39,000 m^3^) that is 8–32 m high above a location equivalent to the 7 s coal seam. However, the karst collapse column damages the integrity of a 280-m thick stratum that runs from the Ordovician limestone to the roof of the 5 s coal seam, forming a water-conducting channel well. Thus, high-pressure karst water of the Ordovician limestone can interface with the water-bearing stratum of sandstone at the 5 s coal seam roof. The karst collapse column has a long axis of about 67 m and a short axis of about 46 m, with an area of about 2875m^2^. The diameter of its spatial form decreases in a segment from 12 to 14 s coal seams. Most of the soft rocks used as fillings in the karst collapse column have aged and softened, as evidenced by accumulations generated by core drilling and those rushing in the roadway. The specific porosity values for the upper, middle and lower segments were calculated to be 0.21, 0.62 and 0.047, respectively.

Ordovician limestones are buried shallowly in the eastern and northern parts of the field but deep in its west and south. Outside the field, it is a subcrop in direct contact with the Quaternary unconsolidated aquifer. The Ordovician limestones have been determined to be an inter-connecting karst water-bearing body based on field pumping tests and long-term dynamic observations on the Ordovician limestone aquifer. However, the Ordovician limestones have dramatically non-uniform water yield properties. To be specific, some boreholes in the north of the well field have a unit water inrush capacity of 6.59 L/(s m) and a permeability of up to 31.87 m/d; however, some boreholes in the southern part have a unit water inrush capacity of less than 0.01 L/(s m). In normal cases, no direct water-filling relationship is formed between the aquifer and the mine. Owing to the existence of karst collapse column, the Ordovician limestone water is directly channelled into the coal measure strata, making it a direct water supply source for mines. Furthermore, the hydraulic pressure of the Ordovician limestone aquifer is 9 MPa^29^.

### Numerical model construction

**Figure 6:**
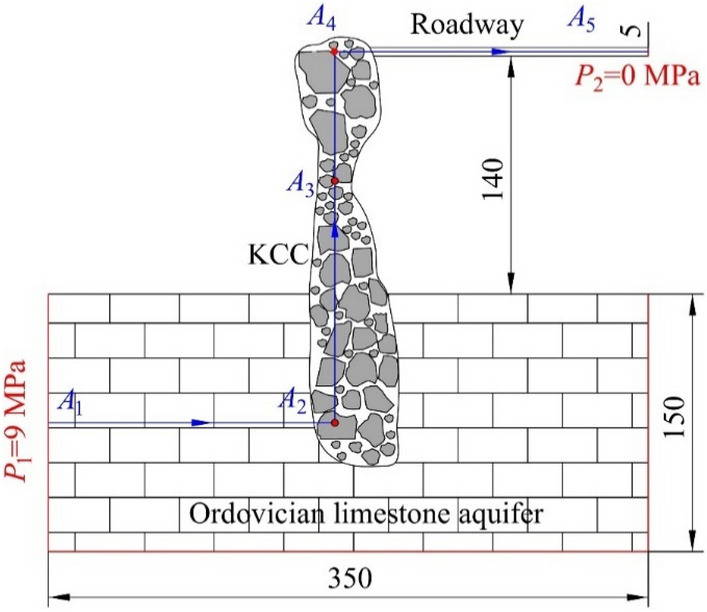
Numerical model of the of the Karst collapse column.

A numerical simulation model for karst collapse column water inrush was developed based on the forms of karst collapse column provided in relevant geological data. The model is composed of three parts. Darcy’s equation was used to express the seepage of the lower Ordovician limestone aquifer, the Forchheimer equations were used to describe the mixed fluid flow of the middle karst collapse column and Navier–Stokes equations were used to describe the upper free flows of roadways. The initial concentrations of migrating particles inside the karst collapse column and the roadway were set at 0.01 and 0, respectively, to account for migrated particle sedimentation in the roadway inside the karst collapse column. Hydraulic pressure of 9 MPa, which is equivalent to that of the Ordovician limestone aquifer, was applied towards the lower part of the model. In addition, the roadway export directly communicated with the air, and the relative pressure was 0. The other exterior boundaries of the model were all confined. Figure 6 depicts the geometry size and boundary conditions of the model. The solution domain was segmented into 35,000 structured grids using triangular elements. Table 3 lists the relevant calculated parameters.

**Table 3:** Mechanical parameters of water rush model.

Parameters	Value	Remarks
*α*_1_	5 × 10^–5^	Obtained by experiment
*α*_2_	2.8	Obtained by experiment
*α*_3_	206.6	Obtained by experiment
*α*_4_	− 8.3	Obtained by experiment
Water density *ρ*_w_ (kg/m^3^)	1000	–
Particle density of filler *ρ*_s_ (kg/m^3^)	2650	Ref.^9^
Viscosity coefficient of water *μ* (Pa s)	1.01 × 10^–3^	–
Initial porosity of Karst collapse column *ε*_0_	0.15	–
Maximum porosity of Karst collapse column *ε*_m_	0.45	–
Aquifer permeability *k*_D_ (m^2^)	3.8 × 10^–11^	–
Average particle size $\overline{d}$ (m)	0.05	Ref.^31^
Talbol power exponent *n*	0.5	Ref.^20^
Underground erosion coefficient *λ* (m^−1^)	30	Ref.^22^
Deposition coefficient *K*_dep_ (s^−1^)	0.01	Ref.^22^

### Result analyses

#### Spatio-temporal evolution law of pressure

**Figure 7:**
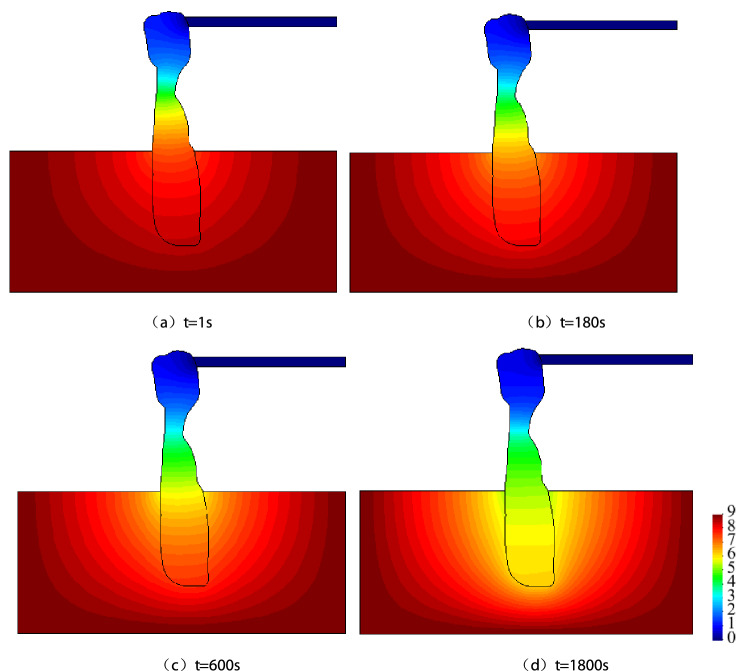
Space–time evolution process of pressure (unit: MPa).

Figure 7 depicts the spatio-temporal evolution process of hydraulic pressure for karst collapse column water inrush in the Fangezhuang Mine. Hydraulic pressure distribution in the aquifer and karst collapse column may change noticeably at different stages of water inrush. Such a phenomenon mainly occurs in the lower part of a karst collapse column and its neighbouring aquifer. In addition, the pressure decreases to the greatest extent at the karst collapse column, but its decline rate becomes gradually small and even remains unchanged in positions far from the karst collapse column. Therefore, we selected hydraulic pressure variations observed in the Ordovician limestone aquifer as an early warning of water inrush.

**Figure 8:**
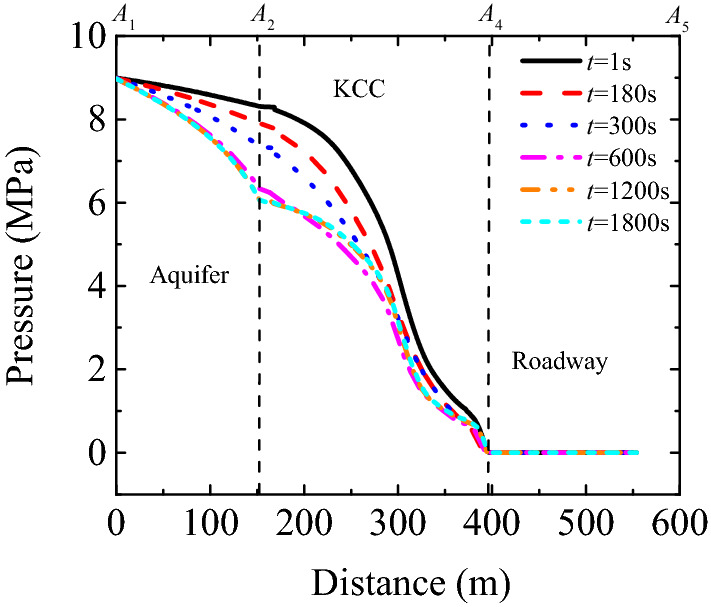
Curve of water pressure from Ordovician limestone aquifer to tunnel (*A*_1_-*A*_2_-*A*_4-_*A*_5_).

The curves in Fig. 8 represent variations in pressure distribution from the Ordovician limestone aquifer to the roadway. As shown in this figure, as the time of water inrush progresses, the pressure on a contact position between the aquifer and the karst collapse column changes significantly, decreasing by 2.19 MPa from 8.31 MPa at the beginning to 6.12 MPa. At other locations, the pressure variation range is relatively narrow. The pressure distribution curves are consistent at t = 1200 s and 1800s, indicating that the fluid pressure field distribution in the karst collapse column reached a steady state. Therefore, hydraulic pressure constantly changes in the entire flow region during water inrush.

#### Spatio-temporal evolution law of flow velocity

**Figure 9:**
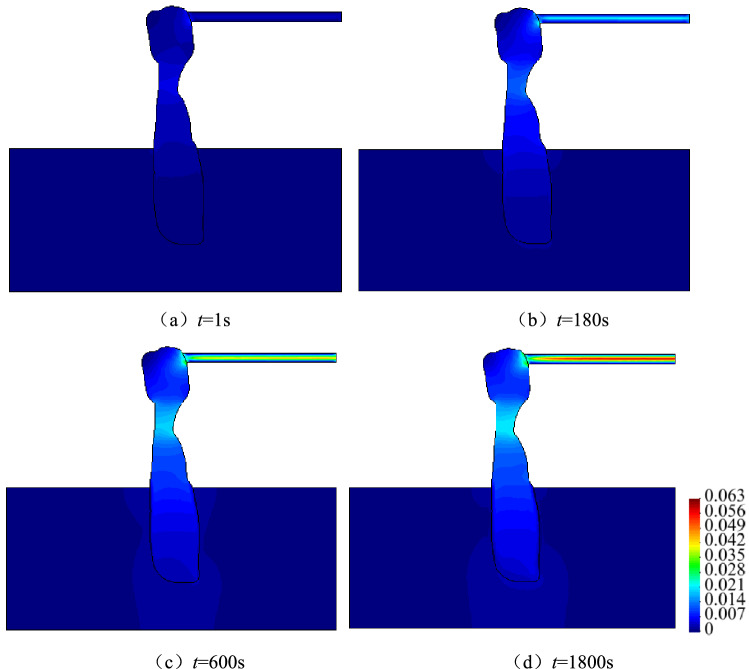
Space–time evolution process of velocity (unit: m/s).

**Figure 10:**
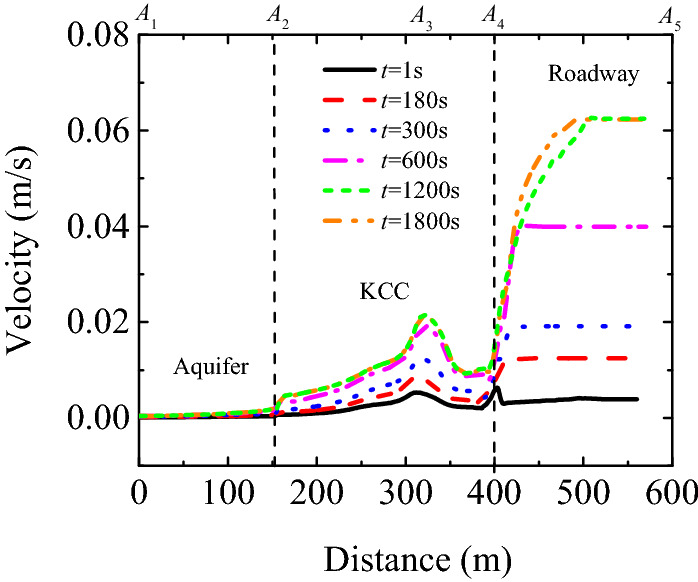
Curve of velocity of flow from Ordovician limestone aquifer to tunnel (*A*_1_-*A*_2_-*A*_4-_*A*_5_).

Figure 9 presents the spatio-temporal evolution of the flow velocity, whereas Fig. 10 depicts the flow velocity distribution curves from the Ordovician limestone aquifer to the roadway. As can be observed, there is a change in the velocity of fluids due to water inrush flood from the aquifer to the roadway through the karst collapse column. According to the spatial analysis results, the flow velocity increases from 4.32 × 10^−4^ to 1.99 × 10^−3^ m/s in a segment from A_1_ to A_2_ of the aquifer (*t* = 1800s), thereby becoming 4.6 times greater. After the fluids flow into the karst collapse column, the decompression action of this column causes a hydraulic disturbance in the aquifer. Consequently, the seepage balance state of the original aquifer is destroyed, and the flow velocity abruptly increases in a short time by an order of magnitude from 1.99 × 10^−3^ to 2.15 × 10^−2^ m/s at the narrowest location of the karst collapse column (i.e. A_3_). This is an early warning of a sudden increase in the flow velocity of the karst collapse column during a transient process of water inrush. Once it enters the roadway, the flow velocity further increases to 6.25 × 10^−2^ m/s. The flow velocity is two orders of magnitude higher than that at A_1_ (4.32 × 10^−4^ m/s), which is the export boundary of the aquifer. In addition, the flow velocity is transformed from uniform changes to ‘step changes’. According to the temporal analysis results, the steady flow velocity is 3.90 × 10^−3^ m/s in the roadway at *t* = 1 s, but it increases to 6.25 × 10^−2^ m/s at *t* = 1,800 s, which is a 16-fold increase. Therefore, karst collapse column water inrush can be regarded as a dynamic process in which seepage changes gradually at first before changing abruptly, and ‘step changes’ increases in flow velocity are the most visual reflection of water inrush occurrence and development.

#### Spatio-temporal evolution law of porosity

**Figure 11:**
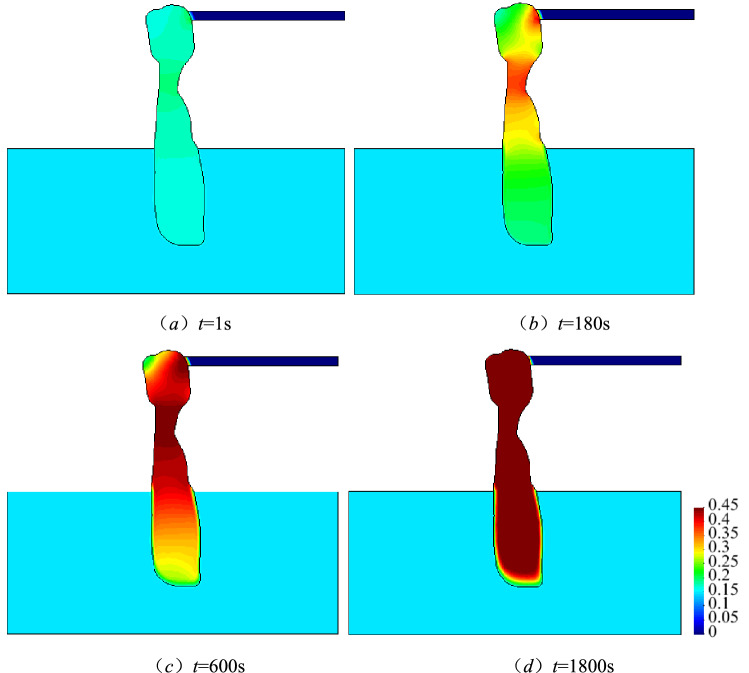
Space–time evolution process of porosity.

**Figure 12:**
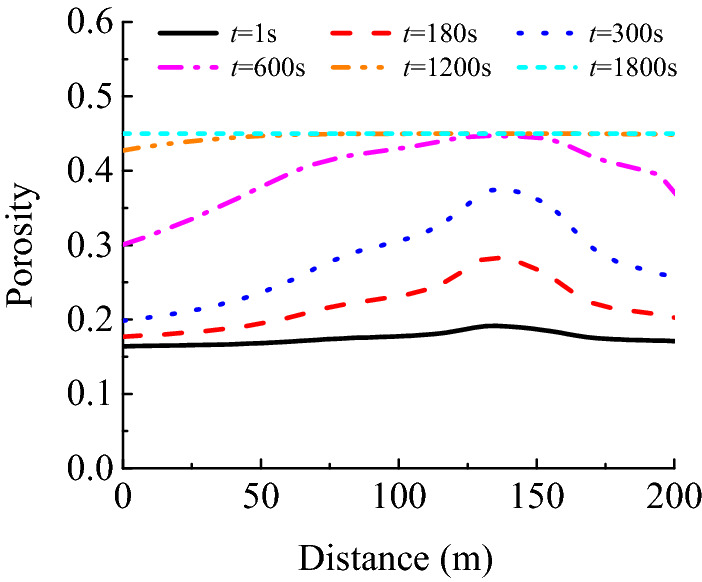
Porosity distribution of measuring line *A*_2_*A*_4_.

Figure 11 depicts the spatio-temporal evolution of porosity within the karst collapse column. Figure 12 shows that the porosity changes along with time along the *A*_2_*A*_*4*_ surveying line. Spatial analysis revealed that the porosity does not change uniformly. Internally filled small particles are activated at the narrowest site of the karst collapse column and its export, resulting in the formation of fluidised particles. The porosity of the above two positions increases sharply as sand-carrying water flows into the roadway. Moreover, their interconnection results in the formation of a preferential migration passage, decreasing the resistance to sand-carrying flows. Thus, conditions beneficial for flow acceleration can be created, enabling high-velocity flows, which facilitate the migration of large particles. This is an interactive variable mass process. The preferred migration passage continues expanding towards the lower part of the karst collapse column until all fine particles are lost and the porosity reaches its peak value. The temporal analysis results revealed that the porosity variations are minor at an initial time of *t* = 1 s. The preferred seepage channel had a prototype form at *t* = 180 s. However, the preferred seepage channel was noticeably formed at *t* = 600 s. Since then, the channel has constantly been expanding circumferentially because of the action of heavy water flows inside it, resulting in the initiation of particles that had been stationary. At *t* = 1800 s, almost all porosity values reach their peaks in the entire karst collapse column. This can be mutually verified with the spatio-temporal evolution law of pressure, velocity and concentration. When the porosity of the karst collapse column increases from 0.16 to 0.45, the Karst collapse colum ‘activates’ and gradually becomes a water-conducting channel, allowing the Ordovician limestone aquifer to communicate hydraulically with the roadway. In this process, the migration of small particles causes a sharp increase in porosity, which is a direct cause of water inrush.

#### Spatio-temporal evolution law of concentration

**Figure 13:**
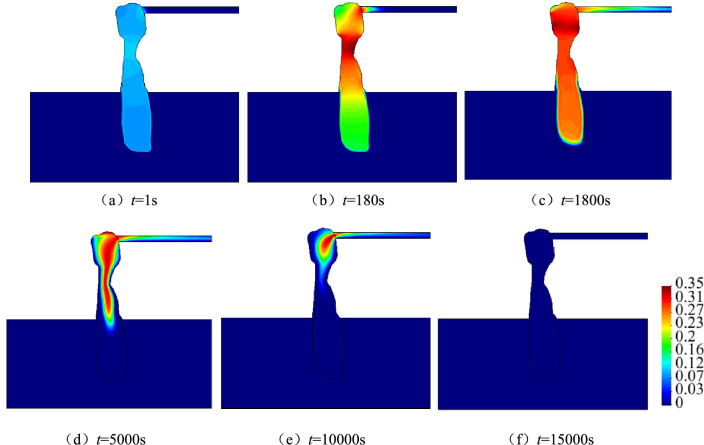
Space–time evolution process of concentration.

**Figure 14:**
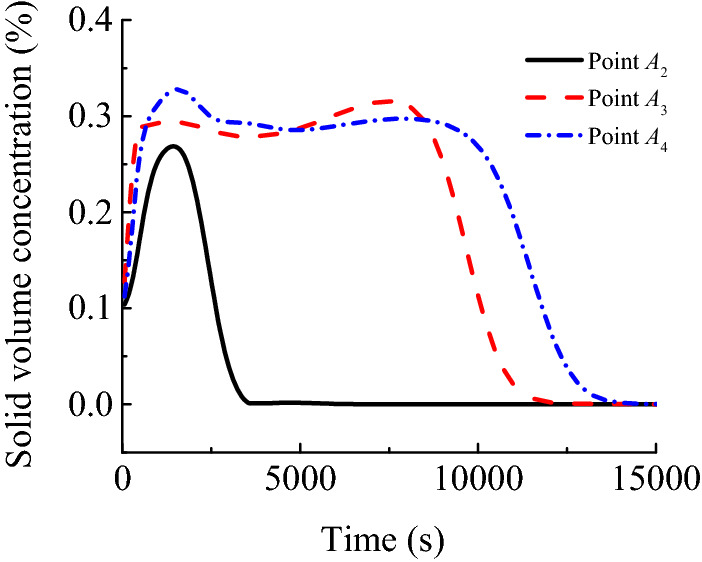
Concentration history curves of different typical points.

Figure 13 illustrates the spatio-temporal evolution of concentrations for migrating particles in a karst collapse column and roadway, whereas Fig. 14 depicts the time-varying concentration curves at typical sites. The concentration has a variation law entirely consistent with that of porosity. Particles at the narrowest site (*A*_*3*_) of the karst collapse column are first fluidised, followed by those at the export (*A*_4_) and the bottom (*A*_2_) of the karst collapse column successively. At the monitoring point (*A*_*2*_), the particle concentration increases rapidly at first to its peak value and then sharply decreases, eventually becoming 0. Only rock skeletons that cannot be fluidised remain there. At *A*_3_ and *A*_4_, the particle concentration increases to its peak and begins to fluctuate around this peak for the following reason. Instead of no particle migration, the particles flowing in have the same volume as the particles flowing out. The migrating particles flood the roadway space as water flows, and their concentration gradually decreases to 0 during sedimentation. Thus, many sediments and rock fragments can be found at the working face and the roadway space after water inrush.

#### Impacts of particle size distribution on karst collapse column water inrush risks

**Figure 15:**
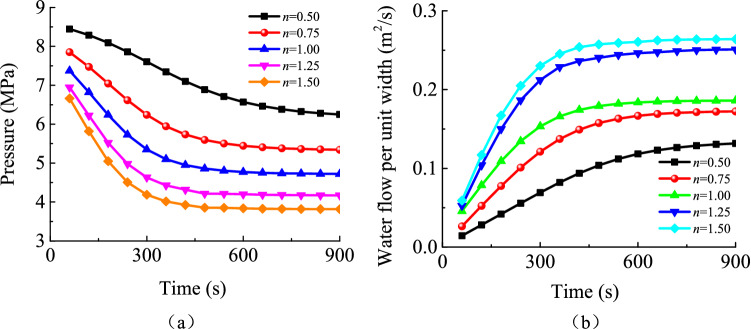
Water inflow and pressure at the entrance of the karst collapse column under different values of *n*: (**a**) pressure; (**b**) water inflow.

**Figure 16:**
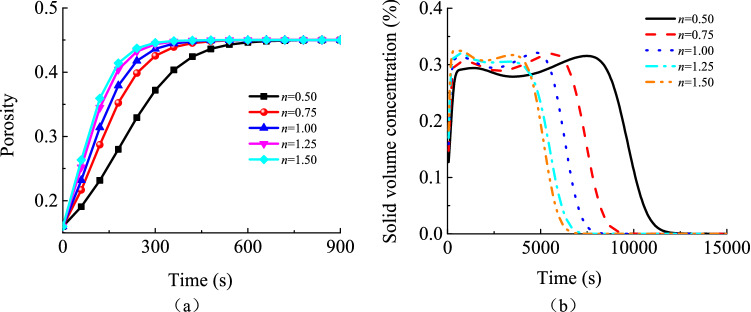
History of porosity and solid volume concentration at monitoring point A_3_ under different values of *n*: (**a**) porosity; (**b**) solid volume concentration.

As shown in Fig. 15, the inlet pressure of a karst collapse column and the flow rate at its export are both very sensitive to particle size distribution. When the hydraulic pressure of an aquifer remains unchanged, the Talbol power exponent increases from 0.5 to 1.5. In this context, the steady hydraulic pressure at the inlet of the karst collapse column decreases by 2.72 MPa from 6.09 to 3.37 MPa. This indicates that an increase in the Talbol power exponent may improve the pressure relief effect as long as the water recharge amount of the aquifer remains constant. At the export of the karst collapse column, the flow rate increases as the Talbol power exponent increases, and the discharge per unit width increases from 0.14 to 0.27 m^2^/s, which is a 93% increase. As shown in Fig. 16, a high Talbol power exponent indicates that the water-percolating capacity of the karst collapse column is high. In addition, particles may be lost more rapidly, resulting in a dramatic increase in the particle concentration in the mixed fluid. The formation of a steady seepage channel with a high porosity also takes a relatively short time, and the water inflow reaches its peak value quickly. The time is shortened by 20% from 720 to 420 s. In such situations, the risk of water inrush becomes more dramatic. Thus, variations in particle size distribution are believed to have a significant effect on variations in water inrush pressure and flow rates at the export in karst collapse columns that are transition areas for aquifer seepage and free flows of roadway water inrush. In practice, water inrush is caused by karst collapse column ‘activation’, which is incurred by particle migration and loss under actions of sufficient water recharge to the aquifer and constant high hydraulic pressure.

A non-linear parameter denoted as *E* was introduced and expressed as follows to quantitatively describe the non-linear flow characteristics of fluids in a karst collapse column^32^:

15 $$E = \frac{{\beta \rho v^{2} }}{{\frac{\mu }{k}v{ + }\beta \rho v^{2} }}$$

**Figure 17:**
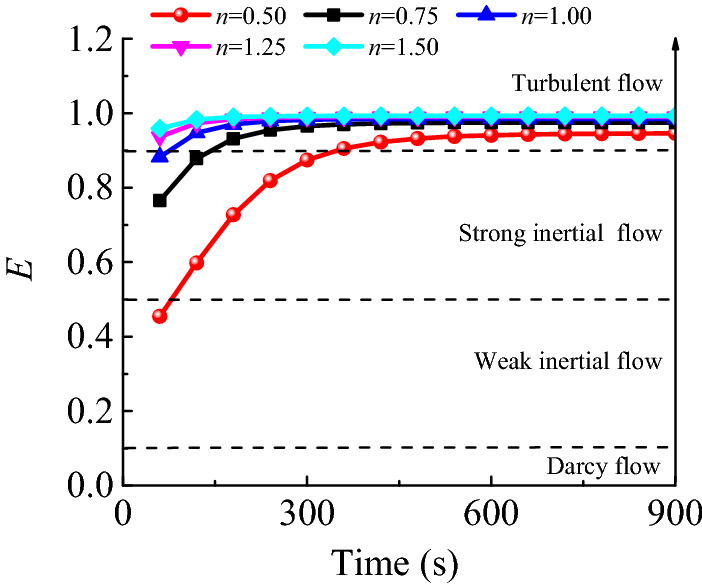
History of *E* at monitoring point *A*_3_ under different values of *n.*

Equation (15) expresses the ratio of a pressure drop caused by inertia force to another pressure drop caused by both inertia force and viscosity. Generally, *E* = 0.1 is selected as the critical point at which the linearity of a fluid flow becomes non-linearity^33^. Shi^22^ emphasised that *E* should be 0.5, which is the critical point at which weak inertial flows change into strong ones, while *E* = 0.9 can be used as the critical point at which strong inertial flows become turbulent flows. As shown in Fig. 17, the curve can be roughly divided into three segments based on the *E* values: (1) weak inertial flows when 0.1 ≤ *E* < 0.5, (2) strong inertial flows when 0.5 ≤ *E* < 0.9 and (3) turbulent flows when 0.9 ≤ *E* < 1.0. Provided that the hydraulic pressure of the aquifer remains constant, a karst collapse column gradually evolves into a highly porous and strongly permeable water-conducting channel during fine particle migration. The flow state of fluids in the column does not remain unchanged; instead, it gradually transforms from a weak inertial flow to a strong inertial flow, eventually becoming a turbulent flow. A seepage model based on a single flow state cannot reflect the nature of water inrush flow state transitions. The Forchheimer equations can effectively represent the intermediate state for water flows changing from a Darcy flow in an Ordovician limestone aquifer to a turbulent flow in the roadway. Moreover, these equations have the potential to quantitatively reveal the flow state transition mechanism caused by particle loss during the entire karst collapse column water inrush process. In the flow region of the karst collapse column, the non-linear parameter (*E*) is above 0.4, which is significantly greater than the critical value of linear flows (i.e. 0.1). Thus, the model built in this study highlights the concept of non-linear flows.

## Conclusions

1. The Talbol power exponent *n* is introduced to properly modify and improve the traditional computational formula, which is $k = 5 \times 10^{ - 5} \frac{{\varepsilon^{3} }}{{(1 - \varepsilon )^{2} }}\overline{d}^{2} n^{2.8}$ and $\beta = 206.6\frac{(1 - \varepsilon )}{{\varepsilon^{3} \overline{d} }}n^{ - 8.3}$.
2. When the hydraulic pressure of an aquifer remains unchanged, the Talbol power exponent increases from 0.5 to 1.5. In this context, the steady hydraulic pressure at the inlet of the karst collapse column decreases by 45% from 6.09 to 3.37 MPa. And the discharge per unit width increases from 0.14 to 0.27 m^2^/s, which is a 93% increase. The formation of a steady seepage channel with a high porosity also takes a relatively short time, and the water inflow reaches its peak value quickly. The time is shortened by about 42% from 720 to 420 s.
3. During the water inrush, the flow state of fluids in karst collapse column gradually transitions from a weak inertial flow to a strong one, eventually becoming a turbulent flow. The Forchheimer equations can reveal the intermediate state of water flows transitioning from Darcy flows in an Ordovician limestone aquifer to turbulent flows in a roadway. Additionally, the equations have the potential to quantitatively uncover the flow state transition mechanism caused by particle losses throughout the entire karst collapse column water inrush process.

## Data Availability

The datasets used and/or analysed during the current study available from the corresponding author on reasonable request.
